# Effect of Underwear Materials on the Thermal Insulation of Barrier Protective Clothing

**DOI:** 10.3390/ma19010124

**Published:** 2025-12-30

**Authors:** Magdalena Młynarczyk, Joanna Orysiak, Aleksandra Kopyt, Szymon Ordysiński

**Affiliations:** 1 Laboratory of Thermal Load, The Department of Ergonomics, Central Institute for Labour Protection—National Research Institute, Czerniakowska St. 16, 00-701 Warsaw, Poland; joanna.orysiak@ciop.pl (J.O.); alkop@ciop.pl (A.K.); 2 Laboratory of Working Conditions Research, The Department of Safety and Health Management, Central Institute for Labour Protection—National Research Institute, Czerniakowska St. 16, 00-701 Warsaw, Poland; szord@ciop.pl

**Keywords:** underwear, thermal comfort, barrier coverall, COVID-19, PPE

## Abstract

Medical personnel wearing barrier clothing protecting against infectious agents are at risk of heat stress resulting from limited heat exchange with the environment. The aim of the study was to assess the impact of changing underwear on the thermal parameters of protective clothing sets and on the expected safe working time. The study used a Newton thermal manikin to determine the thermal insulation and water vapor resistance of clothing sets consisting of three types of underwear (standard medical underwear and short and long thermal underwear) worn under two types of barrier suits. The obtained data were used to conduct physiological simulations in the Predicted Heat Strain (PHS) program, estimating the time it takes for core body temperature to rise to 38 °C in conditions of 22 °C and 35 °C. The results showed that replacing medical underwear with thermal underwear at 22 °C extended safe working time by 24%. In hot conditions (35 °C), the positive impact was smaller, extending working time by a maximum of 4%. Changing the inner layer is an effective method of improving comfort and safety in barrier clothing, especially in thermoneutral conditions.

## 1. Introduction

The heat load of medical workers is the result of three factors: environmental conditions (especially high temperature and high relative humidity), metabolic rate, also related to the work performed, and the protective clothing used.

Environmental conditions in many cases (e.g., operating room, patient room, staff rooms, sterilization room) are characterized by high temperatures. Examples collected by country and room type are included in Table 1.

The data in Table 1 indicates that, in many cases, medical professionals are exposed to high temperatures in the workplace.

Another element of heat stress is metabolic rate, according to Smagowska et al. [10], in selected research areas (operating room and sterilization room), assuming low physical activity (up to 100 W/m^2^). Workload intensity among medical personnel varies depending on the type of work performed. During surgery in the operating room, an anaesthesiologist’s work is characterized by physical activity levels of 70 W/m^2^, while the work of surgical assistants and nurses is 100 W/m^2^, and the work of a surgeon is defined as 130 W/m^2^ [11]. In simulation studies, Potter et al. [12] assumed a workload intensity of two METs (~112 W/m^2^) for nurses.

Often, in the case of medical workers, clothing is intended to protect against infectious agents or pathogens. It is a barrier garment that hinders heat exchange between the human body and the external environment. The thermal parameters describing a set of clothing are thermal insulation (thermal resistance) (R_ct_; the unit of thermal insulation is 1 clo = 0.155 m^2^ K/W) and water vapor resistance (R_et_; the unit is m^2^ kPa/W). The higher the R_ct_ and R_et_ values, the more clothing creates an additional burden on the wearer and hinders heat exchange, thus increasing the risk of excessive thermal stress. According to Smagowska et al. [10], only with clothing with low effective thermal insulation (I_cl_ ~ 0.56 clo; a set of surgical clothing without a gown) can workers experience thermal comfort. However, with clothing with higher barrier properties and increased physical activity, this feeling of comfort is impossible.

For comparison, a set of clothing used during surgical procedures (e.g., a set of surgical clothing + Comfort Plus gown) has a thermal insulation value of approximately 1 clo [10]. Research conducted by Zwolińska and Bogdan [13] indicates that a disposable cotton gown has thermal insulation at the level of I_cl_ = 1.49 clo, a barrier, reusable gown for standard risk operations has I_cl_ = 1.30 clo, and a barrier, reusable gown for high-risk operations has I_cl_ = 1.41 clo.

Heat transfer depends largely on the ability of sweat to evaporate. The clothing parameter that determines this relationship is water vapor resistance. According to the study “Breathable or Vapor-Permeable Clothing?” [14], clothing can be classified into three classes based on water vapor resistance test results: 1 (R_et_ ~ 40 m^2^ kPa/W), 2 (20 m^2^ kPa/W < R_et_ ≤ 40 m^2^ kPa/W), and 3 (R_et_ ≤ 20 m^2^ kPa/W). Maklewska [14] demonstrated that the level of water vapor resistance affects the comfort of clothing. The highest comfort is achieved by clothing made from a set of materials with the lowest R_et_ coefficient, meaning one with a water vapor resistance class of 3. A low R_et_ value facilitates the evaporation of sweat.

Personal protective clothing (PPE) of medical staff is designed to prevent infection with pathogens, and the required high level of protection hinders heat exchange through sweat evaporation. This hinders or significantly limits heat exchange between the wearer and the external environment, significantly reducing heat loss. This impacts the productivity and health of PPE wearers in hot weather and increases the risk of heat stress [15,16].

In the era of the past SARS-CoV-2 pandemic, it is known that the most commonly used PPE at that time was the protective coverall. To ensure the required level of protection, these products are most often made of materials that are impermeable to water vapor. Prolonged work in a protective coverall places particular strain on the body, as it protects the entire body from infectious agents while simultaneously acting as a barrier to heat exchange between the body and the environment. Therefore, whenever possible, efforts should be made to reduce the thermal load on employees resulting from the use of protective clothing. The appropriate selection of protective clothing for a specific workstation, based on a risk analysis, is crucial in this regard.

One of the possibilities of assessing the heat load is to simulate changes in internal temperature using, for example, the Predicted Heat Stress (PHS) program [17,18]. The PHS program, based on the EN ISO 7933 standard [19], allows for the simulation of internal temperature changes during exposure to specific weather conditions, taking into account the clothing used and metabolic rate. The program’s input data includes environmental parameters such as air temperature, natural humidity, radiation, and airflow velocity; metabolic rate; clothing thermal parameters such thermal insulation, water vapor permeability coefficient, and the employee’s acclimatization rate. Output data obtained from the PHS program include the safe working time after which the internal temperature reaches 38 °C. The program validation included data from 672 laboratory experiments and 237 field experiments [20]. Lunerova et al. [21] compared the results of studies involving volunteers to the obtained simulated internal temperature courses, in various ranges of air temperature, metabolic rate, and clothing with different air permeability. Lunerova et al. showed that in the case of air-permeable PPE, the PHS program simulates the internal temperature changes very well [21]. Huang and Li [17] also compared results obtained using the PHS program, the EN ISO 9920 standard [22], and experimental studies. Three types of clothing were tested: normal and light clothing (clothing thermal insulation I_cl_ from 0.48 to 1.11 clo, clothing permeability index i_m_ from 0.21 to 0.49), and cold weather clothing (I_cl_ 2.01 clo and i_m_ 0.2). The best comparisons were obtained for light and normal clothing [17]. Kopeckowa et al. [23] also used PHS for air-permeable military NBC suit M2000 (I_cl_ 1.08 clo, i_m_ 0.34) and impermeable chemical protective clothing Tychem F (I_cl_ 1.06, i_m_ 0.03).

To avoid overheating, working conditions in various hospital spaces can be controlled. However, medical workers often perform their duties (wearing protective clothing) outside of buildings where microclimate parameters cannot be controlled (e.g., paramedics). It is then necessary to look for new solutions to reduce the thermal load on users of barrier clothing. One possibility is to reduce the thermal insulation of the entire set of clothing.

The presented research focused on the possibility of reducing thermal insulation by changing the underwear worn under barrier clothing. Therefore, the aim of the study was to assess the impact of changing underwear on the thermal parameters of protective clothing sets and on the expected safe working time.

## 2. Materials and Methods

The effects of different kinds of medical underwear were investigated for the cases described below.

### 2.1. Materials

Three sets of underwear were used for the study, selected through consultation with the medical community: medical underwear (B1), short (B2) and long (B3) thermal underwear, and two types of coveralls protecting against infectious agents: the Cove Micro coverall without covered seams (K1) and the Tyvek^®^ coverall with covered seams (K2). Detailed information on the above-mentioned clothing items, along with a description of the materials used in their construction, is included in Table 2.

### 2.2. Equipment

#### 2.2.1. Thermal Manikin

**Figure 1:**
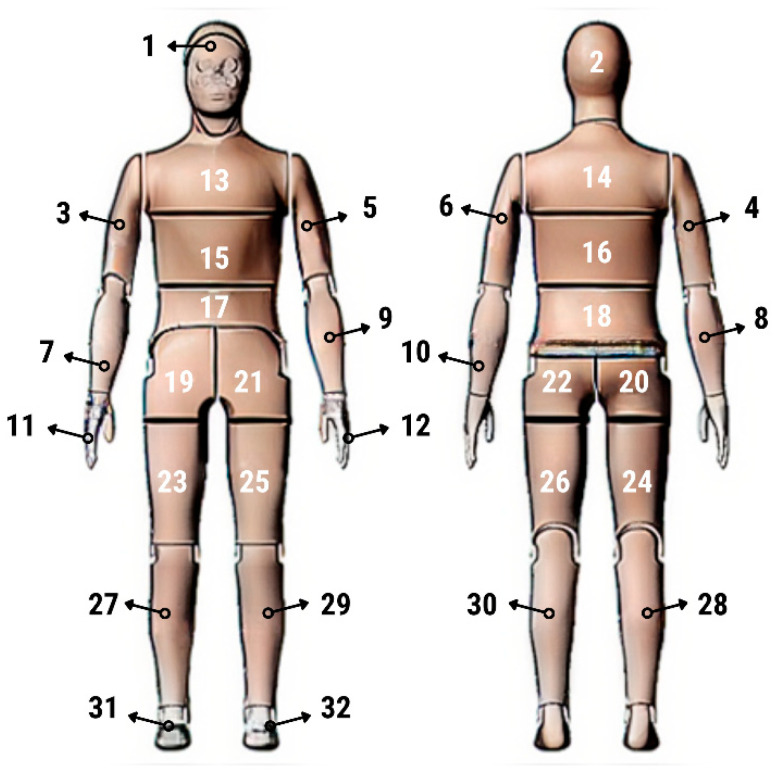
Thermal manikin with scheme of manikin’s segments. (1—Face; 2- Head; 3—R Up Arm Fr; 4—R Up Arm Bk; 5—L Up Arm Fr; 6—L Up Arm Bk; 7—R Forearm Fr; 8—R Forearm Bk; 9—L Forearm Fr; 10—L Forearm Bk; 11—R Hand; 12—L Hand; 13—Upper Chest; 14 —Shoulders; 15—Stomach; 16—Mid Back; 17—Waist, 18—Lower Back; 19—R Up Thigh Fr; 20—R Up Thigh Bk; 21—L Up Thigh Fr; 22—L Up Thigh Bk; 23—R Lwr Thigh Fr; 24—R Lwr Thigh Bk; 25—L Lwr Thigh Fr; 26—L Lwr Thigh Bk; 27—R Calf Fr; 28—R Calf Bk; 29—L Calf Fr; 30—L Calf Bk, 31—R Foot; 32—L Foot.)

Thermal parameters were obtained by the Newton thermal manikin (Measurement Technology Northwest, Seattle, WA, USA). The full-size male thermal manikin consists of 32 active, independently controlled segments. It allows for the simulation of both dry (thermal insulation) and wet (water vapor resistance) heat transfer [25,26,27,28,29]. A general schema of the thermal manikin’s segmentation is shown in Figure 1.

A constant surface temperature mode is one of the manikin’s operating modes.

#### 2.2.2. Climatic Chamber

The tests were performed in the climatic chamber (Weiss, Buchen, Germany, type WK23′), in which air temperatures from −40 °C to +70 °C can be simulated. The chamber’s design allows for the control of values such as relative humidity and air velocity in the horizontal direction (with the air blowing directly to the front of the manikin) [27,30].

#### 2.2.3. Microclimate Meters

**Figure 2:**
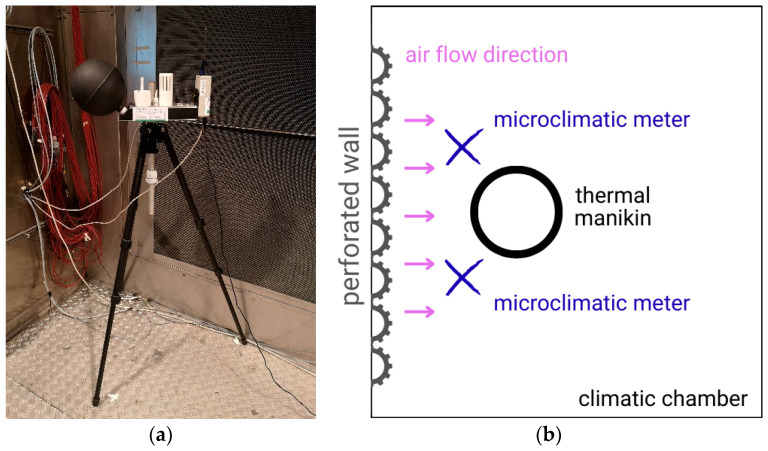
(**a**) The microclimatic meter (MM101); (**b**) Scheme of the meter position during testing.

During the tests of thermal parameters of clothing, two microclimate meters were used to control the conditions inside the climatic chamber: EHA MM101 (Ekohigiena, Środa Śląska, Poland) (Figure 2a) and INNOVA 1221 (LumaSense Technologies, Ballerup, Denmark).

Microclimate meters were placed in front of the thermal manikin according to scheme shown in Figure 2b.

### 2.3. Methodology and Test Variants

#### 2.3.1. Thermal Insulation

Thermal insulation tests were conducted in accordance with the provisions of the EN 342 [31] and EN ISO 15831 [32] standards. Dry heat transfer (thermal insulation) tests were conducted for the selected clothing sets under static conditions (total thermal insulation I_t_).

The tests of thermal parameters of clothing using thermal manikin are described in detail in standards [22,32,33] and in scientific articles [34,35,36,37,38,39].

According to the standards [31,32], clothing thermal insulation tests (static conditions) are conducted in a climatic chamber using a stationary (hanging) thermal manikin. Thermal insulation is measured using the manikin’s operating mode–heat exchange while maintaining a constant manikin surface temperature of 34 °C, with variable power delivered to each segment of the manikin (depending on the clothing set being tested).

Calculating the total clothing thermal insulation R_ct_ = I_t_ was performed by the parallel model (Equation (1)) [32,34].

I_t_ = [(t_s_ − t_a_) × A]/H_c_] [m^2^ K/W], (1)

t_s_ = ∑f_i_ × t_si_ [°C], (2)

H_c_ = ∑H_ci_ [W] (3)

where t_si_—the manikin surface temperature of i^th^ segment [°C]; t_a_—the air temperature [°C]; H_ci_—the observed dry heat loss at i^th^ segment [W]; A—total surface area of the manikin [m^2^]; f_i_—surface area coefficient; t_s_—mean surface temperature of the manikin [°C]; H_c_—the total heating power supplied to the manikin [W].

According to the EN 342 standard [31], the value calculated using the parallel method, i.e., the value calculated as a value related to the entire manikin, should be considered for further evaluation. Between tests conducted on a single set of garments, the EN ISO 15831 standard [32] allows for an error of 4% (for both calculation methods). Kuklane et al. (2012) [40] also indicate that the parallel method provides results that better reflect real conditions. The EN 342 standard [31] recommends using reference values obtained with the parallel method.

#### 2.3.2. Water Vapor Resistance

According to the ASTM F2370 standard [33], water vapor resistance (R_et_) tests are conducted while maintaining a constant surface temperature of the manikin. According to the EN ISO 15831 standard [32], the manikin’s shell temperature is 34 °C. Using the ThermDAC8 computer program (Thermetrics, Seattle, WA, USA), it is possible to control the fluid flow (volume) on individual segments. Water vapor resistance is determined by calculating heat losses (Equation (4)).

R_et_ = [(P_s_ − P_a_) × A]/[H_e_ − (t_s_ − t_a_) × A/R_ct_] [m^2^ kPa/W] (4)

where P_s_—water vapor pressure at the manikin’s sweating surface [kPa]; P_a_—water vapor pressure in the air flowing over the clothing [kPa]; H_e_—power required for sweating areas [W]; A—total area of manikin’s surface that is sweating [m^2^]; R_ct_ = I_t_—total thermal insulation [m^2^K/W].

The above-mentioned standard allows for an error of 10% between tests conducted on a single set of clothing. Tests can be conducted under isothermal conditions or under non-isothermal conditions, as a simulation of real-world conditions. In the presented tests, a sweating intensity of 300 mL/(h·m^2^) was used and conducted under non-isothermal conditions, using a thermal manikin dressed in artificial skin.

#### 2.3.3. TEST VARIANTS

The selected clothing sets were worn on the Newton thermal manikin dressed in specialized skin. Table 3 presents the scope of the tests performed.

At least 2 replicates of thermal insulation and water vapor resistance were performed for each test variant. For R_ct_ tests to be performed correctly, the test in duplicate must not differ by >4%; if this occurs, a third repetition is performed [32]. In the case of R_et_, the permissible value is a 10% difference between repetitions [33].

A detailed description of the clothing components used is provided in Section 2.1, while a description of the research equipment used is provided in Section 2.3.

#### 2.3.4. Thermal Load Simulation—Predicted Internal Body Temperature Changes According to PHS Program

Based on the results of thermal parameters, according to the Predicted Heat Strain program (PHS) [19], the internal body temperature changes during exposure were simulated in established environmental conditions. The program allows for simulations of the internal temperature changes, taking into account also the clothing used and the metabolic rate. The PHS program allows for the input of data:

- Environmental parameters: air and radiation temperature, relative humidity, and air velocity;
- Metabolic rate;
- Clothing thermal parameters: thermal insulation, water vapor resistance, permeability index;
- Acclimatization level of employee.
- The output data obtained from the PHS program were the values of the safe operating time after which the internal temperature reached 38.0 °C (T1) and 38.5 °C (T2).

Based on the results of tests using a thermal manikin, the static clothing permeability index (i_m_) necessary for the simulation was calculated for the tested sets of medical clothing, according to Equation (5) [21,22]:

i_m_ = I_t_/(LR × R_et_) (5)

where I_T_—total thermal insulation [m^2^ K/W], R_et_—water vapor resistance [m^2^Pa/W], LR—Lewis constant (LR = 16.5 K·kPa^−1^).

The i_m_ coefficient is dimensionless and takes values from 0 to 1. The lower the value, the less permeable the material is to water vapor.

## 3. Results

### 3.1. Thermal Insulation

The results of total thermal insulation tests of medical underwear were presented in Table 4 (designations according to Table 2).

The lowest thermal insulation was found in short thermal underwear (B2), followed by long thermal underwear (B3). Medical underwear (B1) had the highest thermal insulation value among the underwear tested.

The microclimatic conditions for testing the thermal insulation of medical underwear are presented in Table 5.

**Figure 3:**
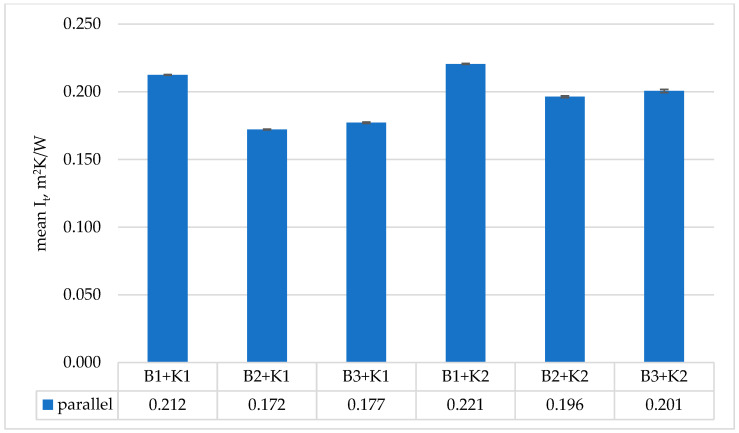
Obtained results of mean total thermal insulation I_t_ (calculated by parallel method) for tested medical sets.

The results of total thermal insulation tests of medical sets were presented in Figure 3 (designations according to Table 2).

The microclimatic conditions for testing the thermal insulation of medical sets were presented in Table 6.

In order to determine significant differences, the percentage difference between the sets was calculated taking into account the study variants (the effect of underwear) (Equation (6)).

Δ = [(value_variant1_ − value_variant2_)/value_variant1_] × 100 [%] (6)

The highest thermal insulation was achieved by the clothing set consisting of medical underwear (B1) and a coverall with hidden seams (K2). Analyzing the results for the sets using thermal underwear and the coverall with leaky seams (K1), the use of short thermal underwear (B2) resulted in this set having the lowest thermal insulation. However, the results obtained for the sets using thermal underwear were within the measurement error range (difference <4%). The difference between the use of medical underwear and thermal underwear in the set was within 7–10%. In the case of the K2 coverall, the lowest values of thermal insulation were obtained when using medical underwear. However, the results obtained for the sets using thermal underwear were within the measurement error range (difference <4%). However, the difference between the use of medical underwear and thermal underwear in the set was in the range of 9–11%.

### 3.2. Evaporative Resistance

The results of the water vapor resistance tests of medical clothing sets were presented in Table 7.

The microclimatic conditions for conducting water vapor resistance tests for the medical clothing sets were presented in Table 8.

The percentage difference between variants was calculated [according to formula (6)]. The highest water vapor resistance was observed for the clothing set consisting of medical underwear and coverall K1. The lowest values were recorded for the thermal underwear set (B3) and the coverall with sealed seams (K2) (difference ~41%). For the K1 coverall, the results obtained for the sets with thermal underwear (B2 or B3) were within the measurement error range (difference <10%). However, the difference between the use of medical underwear and thermal underwear in the set was 23–27%. For the K2 coverall, the results obtained for the sets with thermal underwear (B2) and medical underwear (B1) were within the measurement error range (difference <10%). However, the difference between the use of medical underwear (B1) and short thermal underwear (B3) in the set was 22%.

### 3.3. Results of PHS Simulations

#### 3.3.1. Permeability Index i_m_

The i_m_ values for the clothing sets were calculated, based on the results obtained from tests using the Newton thermal manikin. The obtained values were presented in Table 9.

The obtained i_m_ values ranged from 0.18 (B1+K1) to 0.25 (B3+K2). The result for the B3+K2 differs from the trend observed for the K2 coverall, so a different approach to calculating i_m_ for the medical sets was proposed. Based on the results of the hot plate tests (in Table 2), i_m_ was calculated for the individual materials from which the test sets were made. The share of individual materials in the sets was then considered as 90% for the suit (according to EN 9920 [21]) and 10% for underwear. The i_m_ values obtained for the sets based on the hot plate calculations were included in Table 10.

#### 3.3.2. Simulated the Internal Body Temperature Changes According to the PHS Program

Based on the data from the research on thermal manikin, the computational simulations of the PHS program were performed. The following user data were used:

- Gender: male,
- Body weight: 70 kg,
- Height: 170 cm,
- Body surface area: 1.8 m^2^.

Simulations were performed for three metabolic rate classes: 100 W/m^2^ (low), 165 W/m^2^ (medium), and 230 W/m^2^ (high) and for two different conditions of air temperature: 22 °C (as thermoneutral temperature) and 35 °C (as very high temperature).

The obtained results of the predictable safe operating time to reach the internal body temperature 38 °C (T1) and 38.5 °C (T2) are presented in Table 11 and Table 12 (for air temperature 22 °C and 35 °C, respectively).

**Figure 4:**
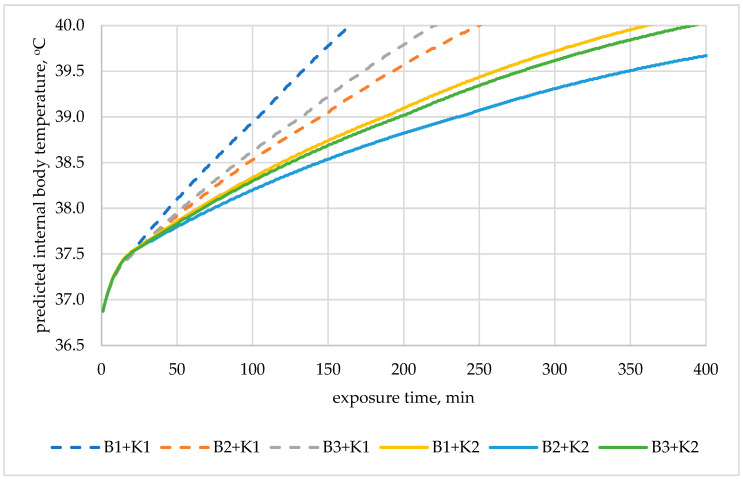
Predicted internal body temperature changes during exposure to 22 °C in the selected medical sets with metabolic rate of 230 W/m^2^_._

Based on the obtained safe exposure times, in the case of an air temperature of 22 °C (1st and 2nd class of metabolism), the use of underwear did not significantly affect the safe working time. For the 3rd metabolic class (230 W/m^2^), the use of underwear varied the obtained safe working time. The predicted internal body temperature changes are shown in Figure 4 (assuming work at 22 °C).

The use of thermal underwear extended the safe working time. In the case of the K1 coverall, switching to thermal underwear meant extending the safe work time (time for increasing internal temperature to 38 °C) by 24% (B2) and 18% (B3) compared to the set with B1 underwear. In the case of the K2 coverall and work at 22 °C, using short thermal underwear (B2) extended the safe work time by 16% compared to the set with B1 underwear.

At a high temperature of 35 °C, there was no significant difference in safe working time values. These values differed only by up to 4% for the 1st metabolic class.

## 4. Discussion

Protective clothing—especially barrier clothing—plays a key role in heat exchange between the human body and the external environment [41]. Depending on the clothing’s thermal parameters, this replacement may be difficult or completely impossible. Therefore, it is important to know the clothing’s thermal parameters and how they affect the various heat transfer pathways [25]. During the work of medics, it is not always possible to control working conditions in terms of air temperature. Therefore, it is important to look for other solutions that would improve work comfort without reducing protection against infectious agents. One such possibility is the use of, for example, phase change material (PCM) vests [35,42] or other structures that absorb heat from the human body [43]. However, these are expensive solutions, sometimes requiring a large investment. Another approach is to change the underlying layer (underwear) [44]. Wang et al. [45] analyzed clothing ensembles with polyester, linen, or cotton inner clothes. Although no significant changes were noted in thermal parameters, significant differences were noted in the volunteers’ comfort responses.

Based on the results of tests using the Newton thermal manikin, it can be concluded that, of the underwear tested, the short thermal underwear (B2) had the lowest thermal insulation value (~0.65 clo), while the medical underwear (B1) had the highest (~0.85 clo). It should also be noted that the B2 and B3 thermal underwear differed in thermal insulation by approximately 4.7%.

When using a clothing set consisting of the K1 and K2 coveralls and three types of underwear, the set with the B2 underwear had the lowest thermal insulation, while the set with the B1 underwear had the highest. Between the sets with thermal underwear (B2 and B3), their thermal insulation values were within the measurement error of <4%. The use of either the B2 or B3 underwear resulted in a significant difference in thermal insulation values. The highest thermal insulation values were recorded during the research for the medical clothing set consisting of the K2 coverall and underwear B1. It amounted to 0.22 m^2^ K/W. These values were similar to the clothing sets used against the Ebola virus (0.24–0.26 m^2^ K/W) [12].

The water vapor resistance values were recorded during the research in the range 64–72 m^2^ Pa/W for sets with K1 coverall and in the range 49–63 m^2^ Pa/W for sets with K2 coverall. These values were similar to the clothing sets used against the Ebola virus, from 47–93 m^2^ Pa/W for the basic set and Tyvek set, respectively [12].

The water vapor resistance values for the described/examined clothing sets show a similar relationship as in the case of thermal insulation for the K1. The set with B2 underwear had the lowest water vapor resistance, while the set with B1 underwear had the highest. Between the sets with thermal underwear, their water vapor resistance was within the permissible measurement error of <10%. The use of B2 or B3 underwear did not result in a significant difference in water vapor resistance values.

A different situation was observed for the K2 coverall. A negligible difference was observed between the water vapor resistance values for the sets with medical underwear (B1) and short thermal underwear (B2). These values differed by approximately 1%, while the use of long thermal underwear (B3) resulted in a decrease in R_et_ by approximately 21% compared to the set with underwear B2. It should be noted, however, that the measurement for B2+K2 was characterized by the greatest variability (sd 3.8 m^2^ Pa/W). Taking into account the variability for B3+K2 and B2+K2, the difference between the results would be reduced to 14%.

To further illustrate the obtained relationships for sets with thermal underwear, the heat loss fluxes (H_ei_—power required for sweating areas on i^th^ manikin’s segments) for the test variants were compared for individual manikin segments, cumulatively as the corpus (Table 13a), upper limbs (Table 13b), and lower limbs (Table 13c). The compared test variants were conducted under similar thermal conditions. Furthermore, segments such as the hands, face, and feet were not included here to better illustrate the changes that occurred.

In the case of the body segments, a difference is visible between the short thermal underwear (B2) and the K1 and K2 coveralls, indicating that lower water vapor resistance was observed in the upper chest segment for the K2 coverall set. The remaining H_ei_ values were similar. In the case of the long underwear (B3), the water vapor resistance was lower in the shoulders segment compared to the K2 coverall set. For the upper limb segments, lower water vapor resistance was observed in the right forearm back segment for the short underwear. The remaining H_ei_ values were similar. Any differences could be due to the coverall design. However, for the long underwear, lower resistance was observed in five of the eight segments (grouped as upper limbs) for the K2 coverall set (primarily for the shoulders, with similar values observed for the forearms). In the case of the lower limbs, the short thermal underwear (B2) covered only the thigh segments. The differences in the calf’s segments were primarily due to the construction of the K1 and K2 coveralls. Lower water vapor resistance was observed in these segments in the case of the K1 coverall. For the long underwear (B3), the differences between the H_ei_ values on the calf’s segments decreased. The differences presented in the segments for the sets indicate that the differences in the total water vapor resistance values (for sets with B2 and B3 underwear) may be due to the coverall construction, but also to better moisture wicking from the manikin’s surface when using the coveralls.

It should also be noted that the coveralls did not adhere to the manikin’s surface. The coveralls are designed to be spacious, and air spaces are created between the material surface and the manikin, which also influences heat exchange (both dry and wet) [34,46,47,48].

The results of tests conducted using a thermal manikin can be used not only to assess the thermal parameters of clothing [36,49] but also to predict thermal comfort or safe working time [21]. The PHS program is used to simulate the course of internal temperature changes, taking into account many input data, including thermal insulation and, indirectly (through the i_m_ coefficient), water vapor resistance [19]. In large cases, the simulations conducted indicate that changing the internal layer (underwear), and thus reducing the total thermal insulation, has a positive effect on the safe working time, defined as the time in which the internal temperature increases to 38 °C. Safe working time studies were conducted with the participation of volunteers. Marszałek et al. [50] tested barrier clothing against liquid chemical agents. The safe working time in such clothing in a hot environment was 30 min. After clothing modifications, the safe working time increased by up to 64% [50].

In the presented study, when predicting working time using the PHS program, switching from polyester blend underwear to polyester thermal underwear resulted in an extension of the safe working time. Under thermoneutral thermal conditions (22 °C) and a metabolic rate of 230 W/m^2^, the safe operating time for the K1 coverall (with B2) was extended by approximately 24% (to approximately 1 h), while for the K2 coverall and B2 underwear, a 75 min work period would be possible. This is similar to the value obtained in the study by Chaudhari et al. [51]. They conducted research involving volunteers using “aerosol medical” personal protective equipment. The results obtained at a temperature of ~21 °C showed that wearing aerosol personal protective equipment for 2 h led to hyperthermia. Furthermore, tachycardia (heart rate 121 bpm) and dehydration (urine specific gravity 1.02) were observed. The volunteers performed activities with a metabolic rate of three MET (approximately 175 W/m^2^).

In the presented study, under high temperature conditions (35 °C) and a metabolic rate of 100 W/m^2^, the safe operating time did not change significantly depending on the underwear used (time increased by 2–5 min). At a metabolic rate of 165 W/m^2^ and 230 W/m^2^, the difference in safe operating time was in the range 1–2 min (from 20 to 50 min). The obtained results were close to the values of safe working time determined during studies involving volunteers [52] wearing anti-Ebola virus sets. The volunteers performed three MET (approx. 175 W/m^2^) activities for 1 h, at 32 °C. In this study, after 1 h exposure, core temperature was in the range 38.2 °C to 38.9 °C. As presented in this publication simulation, the predicted safety time for similar conditions was in the range 30 min (to reach 38 °C) to 50 min (to reach 38.5 °C).

## 5. Conclusions

The problem of overheating in healthcare workers and the subsequent negative consequences for both themselves and patients is very significant. Due to working in barrier clothing, combined with working in high temperatures, also related to climate change, and the inability to regulate working conditions (e.g., air temperature), this problem will only worsen. Therefore, it is crucial to seek new paths and opportunities to improve their work comfort.

One possible solution is the use of cooling vests (e.g., made of PCM); however, this may affect the functionality of protective clothing and also constitute a considerable financial burden for hospitals. Modifying the thermal insulation of the entire clothing set as a potential way to prevent overheating of medical personnel is a new way to approach the thermal load problem. The presented research results and internal temperature simulations conducted with the PHS program indicate the great potential of such a solution.

The results indicate that changing the inner layer can improve work comfort and extend the safe working time in barrier clothing. The simulation results using the PHS program are consistent with those published in the professional literature and obtained from studies involving volunteers. Therefore, it can be assumed that these results are realistic. The air/working temperature is higher, the safe working time is shorter, and the impact of the inner layer is smaller compared to thermoneutral conditions. However, when working at high temperatures in barrier clothing, other solutions should be considered to remove heat from the worker. To systematize the impact of the inner layer, more research should be conducted on other types of underwear with different material compositions to find the optimal combination. The presented results could therefore be a pre-study for further research.

The authors are also aware of the limitations of the research. The predicted results obtained should ideally be verified against the results of controlled studies involving volunteers. The researchers also identified certain methodological limitations. All measurements should have been conducted under identical conditions so that the partial results obtained (H_ei_) could be directly compared, and the segments in which they were significant could be identified. To address the result requiring further verification (R_et_ for B3+K2), an alternative method for calculating the permeability index i_m_ was proposed. Furthermore, the PHS program also has its limitations. It should be used with great caution for clothing characterized by thermal insulation I_cl_ > 1 clo [19]. The research results published by Lunerova et al. [21] also indicate a better fit for barrier (impermeable) clothing using another model (e.g., the FIALA-based model of thermal comfort (FMTK)). Future research should compare the results from both simulations and studies involving volunteers.

## Figures and Tables

**Table 1:** Mean values with standard deviation of temperature measured in selected hospital rooms in different countries.

Place/Country	Range or Maximal Value of Air Temperature [°C]
patients’ room (Italy) [1]	26–29 °C
different wards at a hospital (Iran) [2]	29.9 °C
dialysis storeroom at a hospital (Iran) [2]	31.5 °C
nurse station (emergency hospitalization) at a hospital (Iran) [2]	30 °C
patient rooms (Malaysia) [3]	23.5 °C
visitors room (Malaysia) [3]	23.2 °C
several departments at a hospital (Malaysia) [4]	25.3–28.2 °C
patients’ room at a hospital (Thailand) [5]	21.8–27.9 °C
visitors’ room at a hospital (Thailand) [5]	22.0–27.1 °C
medical staff room at a hospital (Thailand) [5]	24.1–25.6 °C
operating surgeon (Canada) [6]	18.7–29.6 °C
patients’ room (Poland) [7]	21.9–28.4 °C
nurse station at a hospital (UK) [8]	27.7–29.3 °C.
patients’ room (multi-bad) at a hospital (UK) [8,9]	28.1–28.5 °C
operating room (Poland) [10]	22.5 °C
sterilization room (Poland) [10]	23.1 °C
pathology room (Poland) [10]	27.1 °C

**Table 2:** Medical clothing items used for research.

	Description/Composition *	Photo—Front	Photos—Back
B1	medical underwear: top: 65% polyester; 35% cotton pants: 73% polyester; 25% viscose; 2% spandex surface mass: 175.1 ± 4.0 g/m^2^ thickness: 0.33 ± 0.01 mm air permeability: 93.0 ± 5.5 mm/s thermal resistance: 0.02 ± 0.00 m^2^ K/W water vapor resistance: 2.4 ± 0.4 m^2^ Pa/W	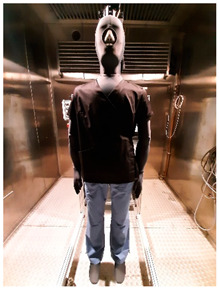	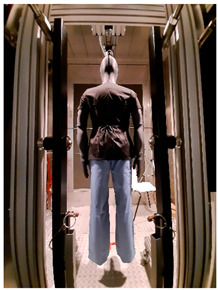
B2	short thermal underwear: 100% polyester surface mass: 197.8 ± 1.9 g/m^2^ thickness: 0.57 ± 0.01 mm air permeability: 652.8 ± 49.4 mm/s thermal resistance: 0.02 ± 0.00 m^2^ K/W water vapor resistance: 2.5 ± 0.1 m^2^ Pa/W	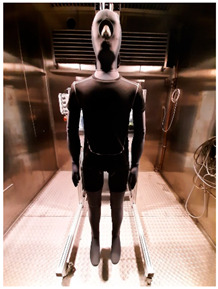	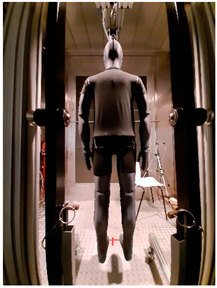
B3	long thermal underwear: 100% polyester surface mass: 242.4 ± 12.5 g/m^2^ thickness: 0.86 ± 0.02 mm air permeability: 111.2 ± 46.2 mm/s thermal resistance: 0.02 ± 0.01 m^2^ K/W water vapor resistance: 3.7 ± 0.5 m^2^ Pa/W	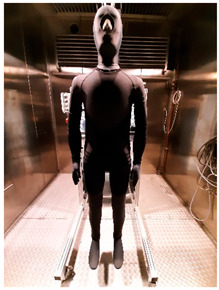	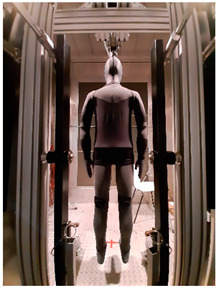
K1	Cove Micro Protective suit/coverall: microporous polypropylene and polyethylene material unsealed seams surface mass: 53.6 ± 2.3 g/m^2^ thickness: 0.32 ± 0.01 mm air permeability: 0.0 ± 0.0 mm/s thermal resistance: 0.02 ± 0.00 m^2^ K/W water vapor resistance: 13.5 ± 3.3 m^2^ Pa/W	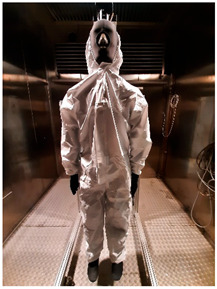	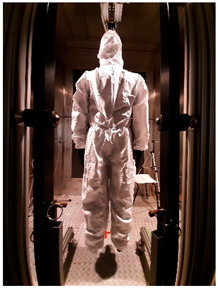
K2	Tyvek 600 Plus protective suit with TY CHA5 T WH 16 socks: Tyvek^®^; seams sealed surface mass: 46.5 ± 2.0 g/m^2^ thickness: 0.31 ± 0.02 mm air permeability: 1.8 ± 0.2 mm/s thermal resistance: 0.02 ± 0.00 m^2^ K/W water vapor resistance: 9.5 ± 1.5 m^2^ Pa/W	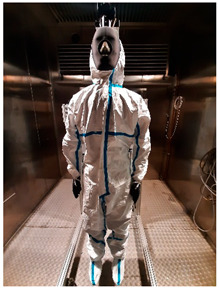	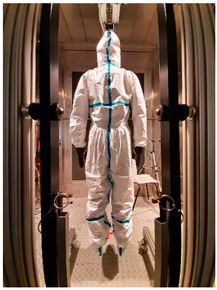

* The values of surface mass, thickness and air permeability obtained as part of the research conducted using laboratory scale, thickness measurement (RAINBOW T; Schroeder Pruftechnik, Germany) and air permeability tester (FX-3300; Textest AG, Switzland); thermal and evaporation resistance values were measured by hot plate (custom-made, Poland) (according to EN ISO 11092 [24]).

**Table 3:** Scope of research (nomenclature according to Table 2).

Outer/Inner Layer	B1	B2	B3
-	R_ct_	R_ct_	R_ct_
K1	R_ct_, R_et_	R_ct_, R_et_	R_ct_, R_et_
K2	R_ct_, R_et_	R_ct_, R_et_	R_ct_, R_et_

**Table 4:** Test results of the total thermal insulation (I_t_) of inner layer/underwear (parallel model).

	Mean I_t_ [m^2^ K/W]	sd
B1	0.132	0.000
B2	0.101	0.000
B3	0.106	0.001

**Table 5:** Microclimatic conditions for testing the total thermal insulation of medical underwear: t_a_—air temperature, RH—relative humidity, V_a_—air velocity (mean value with standard deviations).

	t_a_ °C	RH %	V_a_ m/s
	Test 1		
B1	20.3 ± 0.0	30.8 ± 0.0	0.43 ± 0.01
B2	20.4 ± 0.0	32.3 ± 0.2	0.47 ± 0.01
B3	18.4 ± 0.0	32.0 ± 0.1	0.45 ± 0.01
	Test 2		
B1	20.3 ± 0.0	30.5 ± 0.1	0.43 ± 0.01
B2	20.4 ± 0.0	31.5 ± 0.2	0.47 ± 0.00
B3	18.4 ± 0.0	31.1 ± 0.2	0.47 ± 0.00

**Table 6:** Microclimatic conditions for testing the total thermal insulation of medical sets.

Variant	t_a_ °C	RH %	V_a_ m/s
	Test 1		
B1+K1	18.3 ± 0.0	31.7 ± 0.1	0.44 ± 0.00
B2+K1	18.3 ± 0.0	36.1 ± 0.1	0.43 ± 0.00
B3+K1	18.3 ± 0.0	34.7 ± 0.0	0.43 ± 0.00
B1+K2	15.3 ± 0.0	37.1 ± 0.1	0.45 ± 0.01
B2+K2	15.4 ± 0.0	29.4 ± 0.0	0.45 ± 0.01
B3+K2	15.3 ± 0.0	33.8 ± 0.1	0.44 ± 0.01
	Test 2		
B1+K1	18.3 ± 0.0	31.8 ± 0.0	0.44 ± 0.01
B2+K1	18.3 ± 0.0	36.3 ± 0.0	0.44 ± 0.01
B3+K1	18.3 ± 0.0	34.5 ± 0.1	0.43 ± 0.00
B1+K2	15.3 ± 0.0	36.6 ± 0.1	0.46 ± 0.01
B2+K2	15.3 ± 0.0	29.5 ± 0.1	0.44 ± 0.01
B3+K2	15.3 ± 0.0	34.1 ± 0.1	0.43 ± 0.01

**Table 7:** Test results of the water vapor resistance R_et_ of medical sets.

Variant	Mean R_et_ [m^2^ Pa/W]	sd
B1+K1	71.6	0.2
B2+K1	64.7	1.2
B3+K1	66.1	1.0
B1+K2	63.2	1.4
B2+K2	62.4	3.8
B3+K2	49.3	1.0

**Table 8:** Microclimatic conditions for testing the water vapor resistance of medical sets.

Variant	t_a_ °C	RH %	V_a_ m/s
Test 1			
B1+K1	18.3 ± 0.0	67.1 ± 0.1	0.46 ± 0.01
B2+K1	15.3 ± 0.0	65.2 ± 0.2	0.42 ± 0.01
B3+K1	15.3 ± 0.0	55.3 ± 0.9	0.42 ± 0.01
B1+K2	15.3 ± 0.0	58.2 ± 0.3	0.43 ± 0.01
B2+K2	15.3 ± 0.0	50.0 ± 0.8	0.45 ± 0.01
B3+K2	15.3 ± 0.0	57.5 ± 0.3	0.43 ± 0.01
Test 2			
B1+K1	18.3 ± 0.0	67.2 ± 0.1	0.46 ± 0.01
B2+K1	15.3 ± 0.0	66.1 ± 0.2	0.42 ± 0.01
B3+K1	15.3 ± 0.0	59.0 ± 0.1	0.42 ± 0.03
B1+K2	15.3 ± 0.0	59.4 ± 0.2	0.43 ± 0.01
B2+K2	15.3 ± 0.0	53.5 ± 0.6	0.44 ± 0.01
B3+K2	15.3 ± 0.0	58.9 ± 0.2	0.43 ± 0.01

**Table 9:** The values of permeability index (i_m_) for clothing sets (based on the results of tests using a thermal manikin).

Clothing Sets/Variant	i_m_
B1+K1	0.18
B2+K1	0.16
B3+K1	0.16
B1+K2	0.21
B2+K2	0.19
B3+K2	0.25

**Table 10:** The values of permeability index (i_m_) for clothing sets (based on the results of tests using a hot plate).

Clothing Sets/Variant	i_m_
B1	0.50
B2	0.38
B3	0.36
K1	0.11
K2	0.15
B1+K1	0.15
B2+K1	0.13
B3+K1	0.13
B1+K2	0.19
B2+K2	0.17
B3+K2	0.17

**Table 11:** The simulated safety time of work to achieve core temperature: 38.0 °C (T1), and 38.5 °C (T2) in different variants for parameters: t_a_ 22 °C, V_a_ 0.4 m/s, RH 50%, including three classes of metabolic rate: 100 W/m^2^, 165 W/m^2^ and 230 W/m^2^.

Variants		T1, min	T2, min
100 W/m^2^			
K1	B1+K1	n.a.	n.a.
	B2+K1	n.a.	n.a.
	B3+K1	n.a.	n.a.
K2	B1+K2	n.a.	n.a.
	B2+K2	n.a.	n.a.
	B3+K2	n.a.	n.a.
165 W/m^2^			
K1	B1+K1	n.a.	n.a.
	B2+K1	n.a.	n.a.
	B3+K1	n.a.	n.a.
K2	B1+K2	n.a.	n.a.
	B2+K2	n.a.	n.a.
	B3+K2	n.a.	n.a.
230 W/m^2^			
K1	B1+K1	45	73
	B2+K1	56	97
	B3+K1	53	90
K2	B1+K2	64	119
	B2+K2	74	144
	B3+K2	67	125

Note: n.a.—not achieved.

**Table 12:** The simulated safety time of work to achieve core temperature: 38.0 °C (T1), and 38.5 °C (T2) in different variants for parameters: t_a_ 35 °C, V_a_ 0.4 m/s, RH 50%, including three classes of metabolic rate: 100 W/m^2^, 165 W/m^2^ and 230 W/m^2^.

Variants		T1, min	T2, min
100 W/m^2^			
K1	B1+K1	55	81
	B2+K1	57	84
	B3+K1	55	82
K2	B1+K2	69	103
	B2+K2	68	101
	B3+K2	66	98
165 W/m^2^			
K1	B1+K1	30	43
	B2+K1	30	44
	B3+K1	30	43
K2	B1+K2	34	50
	B2+K2	34	50
	B3+K2	34	49
230 W/m^2^			
K1	B1+K1	20	28
	B2+K1	20	29
	B3+K1	20	29
K2	B1+K2	22	32
	B2+K2	22	31
	B3+K2	22	31

**Table 13:** Power required for sweating areas on the i^th^ manikin’s segments (H_ei_) for medical sets.

Variant	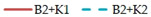	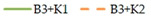
(a) corpus	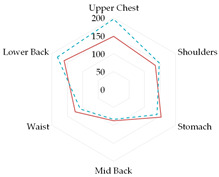	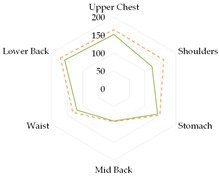
(b) upper limbs	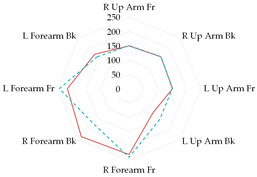	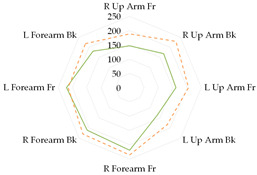
(c) lower limbs	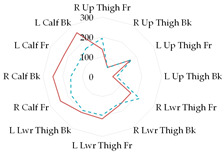	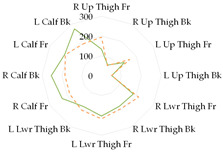

Note: (a) corpus: Upper Chest, Shoulders, Stomach, Mid Back, Waist, Lower Back; (b) upper limbs: Right Upper Arm Front, Right Upper Arm Back, Left Upper Arm Front, Left Upper Arm Back, Right Forearm Front, Right Forearm Back, Left Forearm Front, Left Forearm Back; (c) lower limbs: Right Upper Thigh Front, Right Upper Thing Back, Left Upper Thigh Front, Left Upper Thigh Back, Right Lower Thigh Front, Right Lower Thigh Back, Left lower thigh Front, Left Lower Thigh Back, Right Calf Front, Right Calf Back, Left Calf Front, Left Calf Back.

## Data Availability

The original contributions presented in this study are included in the article. Further inquiries can be directed to the corresponding author.
